# Aortic Aneurism Bursting into the Œsophagus

**Published:** 1831

**Authors:** 


### XXXV.

### Aortic Aneurism bursting into the Œsophagus. By S. Cooper, Esq.

*Case.*—John Backhouse, æt. 38, a muscular man, and axletree maker, applied at the Bloomsbury Dispensary on the 11th Feb. 1830. We cannot abridge Mr. Cooper's short statement, and his observations are so much to the point that we are disposed to insert them.

"The patient had a strongly pulsating tumour, about five inches in diameter, very prominent, situated to the left of the dorsal vertebræ, and extending under the basis of the scapula, which was thrust considerably outwards by it. He complained of palpitations, oppressed breathing, and bloody expectoration. By means of bleeding low regimen, digitalis, purgatives, and quietude, he was so much relieved in three weeks, that he was able to resume his work, which, laborious as it was, he continued regularly, and without difficulty, until the 16th of Sept. when he vomited up suddenly nearly three quarts of blood. He immediately fainted; but revived, and afterwards voided a large quantity of blood from the bowels. On the 27th of September, feeling himself even better than he had been before the loss of blood, he returned once more to his trade, and continued his labour, without interruption, until the 6th of November, when, finding himself indisposed, he remained at home, and sent to the Dispensary for medical assistance. Dr. Richard Pinckard and Mr. Miller, who now visited him, found him much reduced, weak, and languid, with a feeble pulse. They were surprised, however, to find, that the large external tumour was no longer visible, though its pulsations were yet distinguishable, when the fingers were firmly applied to the part.

November 9th. After having vomited up rather less than a pint of florid blood, the patient suddenly expired.

November 11th. The body was opened by Mr. Miller, in the presence of Drs. Pinckard, Mr. C. Griffith, and myself. The heart and lungs were healthy. The aorta was found to have given way, a little below its arch. The aneurismal sac, which was ample, was behind in contact with the dorsal vertebræ and the ribs; but the main portion of it extended between the diaphragm and the left lung, over the edge of which a prolongation was thrown in the form of a tumour, of about the size of a lemon. All the sac, situated under the left lung, and upon its edge, was filled with solid fibrine, arranged in concentric layers, the outer of which were particularly firm, resembling boiled meat. The size of the whole mass of fibrine was equal to that of a half-quartern loaf. Towards the spine, the sac contained blood, some of which was in a fluid state. Here, also, the aneurism had formed an excavation by the absorption of the spinal ends of the sixth and seventh ribs, and of the transverse processes, and a considerable portion of the bodies of three dorsal vertebræ. The left lung, though of a healthy colour and consistence, had evidently been seriously compressed. In the œsophagus, an ulcerated opening, not quite so large as a sixpence, communicated with the cavity of the aneurism. This aperture, through which the fatal loss of blood had occurred, as well as the earlier profuse bleeding, was on a level with the division of the bronchiæ, to which the sac was intimately connected by adhesions. In the stomach, three pounds of coagulated blood were found, besides several ounces of serum, and the duodenum contained another pound of blood.

In this case, the following circumstances seem to me most worthy of note.

1st. The patient's having survived the first bursting of the aneurism from the 16th of September until the 9th of November, when a second copious hæmorrhage took place, and proved fatal. He lived, therefore, nearly eight weeks after a communication had been formed by ulceration between the aneurismal cavity and the œsophagus, and this notwithstanding his having followed, during a considerable part of the time, the laborious occupation of a wheelwright. No doubt, the fainting, induced by the first profuse hæmorrhage, was the means of saving him on that occasion by reducing the force of the heart's action, aud promoting the formation of a coagulum directly over the aperture, that made the communication between the aneurism and the œsophagus.

2dly. The subsidence of the external tumour, following the first copious loss of blood, and, probably, in a great measure, produced by it.

3dly. The displacement of the basis of the scapula by the aneurismal tumour, when the disease was prominent and conspicuous by the side of the spine. I have never seen another aneurism of the aorta that had this effect; but, the particulars of one example of this reremoval of the scapula from its right position, by the protrusion of an aortic aneurism, were transmitted to the Academy of Medicine at Paris, by Dr. Lenoble, of Versailles.

4thly. In the case now related, the pathologist cannot fail to admire the extraordinary efforts made by nature to bring about the cure of the disease. For this purpose, the greater part of the aneurismal cavity had been filled up with successive layers of compact fibrinous matter. In fact, the only portion of the sac, not occupied by this substance, was that towards the vertebræ and the œsophagus."

---

